# 2-Deoxyglucose and Newcastle Disease Virus Synergize to Kill Breast Cancer Cells by Inhibition of Glycolysis Pathway Through Glyceraldehyde3-Phosphate Downregulation

**DOI:** 10.3389/fmolb.2019.00090

**Published:** 2019-09-27

**Authors:** Ahmed Majeed Al-Shammari, Amer Hasan Abdullah, Zainab Majid Allami, Nahi Y. Yaseen

**Affiliations:** ^1^Experimental Therapy Department, Iraqi Centre for Cancer and Medical Genetic Research, Mustansiriyah University, Baghdad, Iraq; ^2^Department of Chemistry, College of Science, Mustansiriyah University, Baghdad, Iraq

**Keywords:** glycolysis inhibition, virotherapy, cancer metabolism, breast cancer model, Warburg effect

## Abstract

Targeting cancer cells metabolism is promising strategy in inhibiting cancer cells progression that are known to exhibit increased aerobic glycolysis. We used the glucose analog 2-Deoxyglucose (2-DG) as a competitor molecule of glucose. To further enhance the effectiveness of 2-DG, the Newcastle disease virus (NDV) was used as a combination virotherapy to enhance the anti-tumor effect. Human and mouse-breast cancer cells were treated by NDV and/or 2-DG. The effect was analyzed by study cell viability, apoptosis and level of glyceraldehyde3-phosphate (GAPDH) by ELISA and QPCR assays. Synergistic cytotoxicity was found after a 72-h treatment of human- and mouse-breast cancer cells with 2-DG in combination with NDV at different concentrations. The synergistic cytotoxicity was accompanied by apoptotic cell death and GAPDH downregulation and inhibition to glycolysis product pyruvate. The combination treatment showed significant tumor growth inhibition compared to single treatments *in vivo*. Our results suggest the effectiveness of a novel strategy for anti-breast cancer therapy through glycolysis inhibition and GAPDH downregulation.

## Introduction

The metabolism of cancer cells is glycolysis dependent. Cancer cells continue to rely on glycolysis rather than oxidative phosphorylation even under aerobic conditions, which requires high quantities of glucose to produce energy and support the metabolic function (i.e., the Warburg effect) (Warburg, 1956). This effect means that the glucose uptake of cancer cells is higher than that of normal tissues. Thus, the glucose metabolism could be targeted as a site for chemotherapeutic intervention by agents such as 2-deoxy-D-glucose (2-DG) (Aft et al., 2002). Ciavardelli et al. (2014) showed that breast-cancer stem cells rely on fermentative glycolysis and are sensitive to 2-DG treatment.

The non-metabolizable glucose analog 2-DG blocks the first step in glycolysis. It is phosphorylated by hexokinase to produce 2-DG-6P, which cannot be metabolized. It accumulates in cancer cells because the reverse reaction is also blocked (Barban and Schulze, 1961), leading to depleted cellular ATP, antioxidants, and glycolysis intermediates, which are essential for cell survival and proliferation, thereby causing cell growth arrest and death (Zhang et al., 2014). 2-DG has exhibited a cytotoxic effect in breast cancer cells, especially those with mitochondrial respiratory defects or defects induced by mitochondria-targeted drugs (Cheng et al., 2012). Targeting the cellular metabolism could improve cancer therapeutics (Zhao et al., 2013). 2-DG is one of best-known anti-metabolites in cancer cells (Keenan et al., 2004). It has been described as enhancing the therapeutic efficacy of radiation and chemotherapies against different tumors (Gupta et al., 2009). Aghaee et al. (2012) reported that 2-DG acted as a breast cancer sensitizer to irradiation and chemotherapy, thus improving the treatment and enhancing cytotoxicity via oxidative stress. The gold standard for estimating cell metabolic activity is the analysis of bioenergetic signatures (i.e., the ratio of beta-F1-ATPase and GAPDH) in different tumors. GAPDH plays an essential role in the glycolysis pathway. It has several functions, such as protecting the cell from oxidative stress, inducing cell death, and so forth. Among all other members of the glycolysis pathway, GAPDH is considered one of the most promising therapeutic targets (Krasnov et al., 2013).

Cancer virotherapy using oncolytic viruses was found to be effective in combination with chemotherapies (Yaseen and Al-Shammari, 2014) radiotherapy (Harrington et al., 2010). Because malignant tumors are incurable diseases in general, the research on novel therapeutic strategies, such as oncolytic virotherapy, are advancing rapidly toward clinical trials (Ottolino-Perry et al., 2010). Virotherapy is considered a smart targeted therapy because it selectively enters and destroys cancer cells, without affecting normal tissue. The injected viral dose continuous to amplify inside the tumor mass and replicate until it is stopped by the immune system (Kirn et al., 2001; Al-Shammari et al., 2014a). As a monotherapy, oncolytic virotherapy has not been fully effective in the eradication of tumors in both animal models and clinical studies. For complete tumor destruction, the most effective therapy is to combine the oncolytic virotherapy mechanisms with other novel treatment modalities in the emerging field of gene therapy as well as classical chemo- and radiation therapies (Chu et al., 2004). The strategy of combination therapy is to attack tumor cells through different mechanisms to prevent cancer cells from developing resistance to therapy (Kumar et al., 2008). The avian Newcastle disease virus (NDV), which has been used in virotherapy since the late Twentieth century (Cassel and Garrett, 1965), is known for its selectivity and status as a natural onco-tropism RNA virus (Schirrmacher, 2015). The Iraqi NDV is a virulent strain of avian paramyxovirus of the genus Avulavirus, which belongs to the family Paramyxoviridae (Al-Shammari et al., 2014b). The Iraqi NDV virulent strain was proved to be oncolytic (Al-Shammari et al., 2010). The Iraqi oncolytic strain of NDV was shown to kill tumor cells by several mechanisms, such as the generation of a specific immune response against infected tumor cells (Al-Shamery et al., 2009, 2011) Furthermore, NDV induced apoptosis in the infected cells (Al-Shammary et al., 2014; Al-Shammari et al., 2015a). NDV in combination with 5-Flourouracil, cyclophosphamide, and many others was shown to have synergistic and enhanced anti-tumor effects (Al-Shammari et al., 2011, 2016).

The aim of the present study is to investigate the following: (i) the increased sensitivity of breast cancer cells to oncolytic virotherapy using the glucose inhibitor 2-deoxyglucose (2-DG); (ii) the enhanced cytotoxic effects of synergistic combinations of different concentrations; (iii) the mechanisms of the combination therapies that enhance cytotoxicity.

## Materials and Methods

### Cell Culture

The AMJ13 breast cancer cell line (Al-Shammari et al., 2015b), the AMN3 murine mammary adenocarcinoma cell line, and the rat embryo fibroblast (REF) cell line were cultured in a RPMI-1640 medium with 10% fetal bovine serum (FBS), 100 units/mL penicillin, and 100 μg/mL streptomycin and then incubated at 37°C. A human epithelial carcinoma (Hela) cell line was cultured in MEM medium with 10% fetal bovine serum (FBS), 100 units/mL penicillin, and 100 μg/mL streptomycin and then incubated at 37°C. All the cell lines were supplied by the Cell Bank Unit in the Experimental Therapy Department at the Iraqi Center for Cancer and Medical Genetic Research (ICCMGR). These cells are regularly assessed for standard growth characteristics, and they are regularly authenticated.

### Virus

The Iraqi AMHA1 strain of NDV (Iraq/Najaf/ICCMGR/2013) (Al-Shammari et al., 2014b,c) was supplied by the Cell Bank Unit in the Experimental Therapy Department at the Iraqi Center for Cancer and Medical Genetic Research (ICCMGR). A stock of infectious virus was propagated in embryonated chicken eggs (Al-Kindi Company, Baghdad, Iraq), harvested from allantoic fluid, and then purified of debris by centrifugation (3,000 rpm, 30 min at 4°C). NDV was quantified by a hemagglutination test in which one hemagglutination unit (HAU) was defined as the smallest virus concentration leading to visible chicken erythrocyte agglutination. The aliquoted virus was stored at −80°C. Viral titers were determined by a 50% tissue culture infective dose (TCID50) titration on Hela cells according to the standard procedure (Phuong et al., 2003).

### Combination Cytotoxicity Assays and Chou-Talalay Analysis

The AMJ13, AMN3, and REF cells were seeded at 7,000 cells/well in 96 well plates and incubated overnight. Two-fold serial dilutions of NDV first were added at an MOI of one (7000 TICD50) with 2-fold serial dilutions and then incubated for 2 h at room temperature. The series of 2-fold dilutions of 2-DG then was added (20,800- 10,400- 5,200- 2,600- 1,300- 650 μg/ml) and incubated for 72 h. To measure the growth inhibition rate, cell viability assays were performed after incubation. After 72 h of infection, the cell viability was measured by removing the medium, adding 28 μl of a 2-mg/ml solution of MTT (Bio-World, USA), and incubating for 1.5 h at 37°C. The crystals remaining in the wells were solubilized by the addition of 130 μl of DMSO (dimethyl sulphoxide) (Santa Cruz Biotechnology, USA) and then incubated for 15 min with shaking. Absorbency was determined on a microplate reader (Expert Plus Reader, Asyshitech, Austria) at 492 nm (test wavelength). The assay was performed in triplicate and repeated at least four times. The mean viability of the treated cells for each dilution was calculated as a percentage relative to the control wells treated with media alone (100% survival) ± SEM.

The median effective doses (ED50) the 2-DG and NDV were calculated in each cell line. To determine synergism, NDV and 2-DG were studied as a non-constant ratio. To analyze the combination of NDV and 2-DG, Chou-Talalay combination indices (CI) were calculated using CompuSyn software (CompuSyn, Inc., Paramus, NJ, USA). The non-fixed ratios of NDV and 2-DG as well as mutually exclusive equations were used to determine the CIs. A CI between 0.9 and 1.1 was considered additive, whereas CI < 0.9 and CI > 1.1 indicated synergism and antagonism, respectively (Chou, 2010).

### Assessment of Apoptosis

The Acridine Orange-Propidium iodide (AO/PI) dual staining method was used to measure the apoptotic death of the AMN3 and AMJ13 cell lines. Cells seeded at 7,000 cells/well in a 96-well plate and incubated overnight at 37°C. Cells were treated with to the least synergistic dose of 2DG–NDV for 48 h, all treated and untreated cells were incubated with AO/PI at 20 μg/ml each for 2 min at room temperature before observing and photograph under inverted fluorescent microscope (Leica Microsystems, Germany) at a magnification of ×200 (Ali et al., 2019).

### Quantification of the GAPDH Enzyme

To quantify the GAPDH enzyme level, a cell lysate of cells treated with the least synergistic doses was collected at regular intervals (6, 12, 18, and 24 h) and frozen until the analysis was conducted. The concentration of the GAPDH enzyme was determined by using an enzyme-linked immunosorbent assay (ELISA) kit according to the manufacturer's protocol (US Biological, USA) for a human and mouse glyceraldehyde 3-phosphate dehydrogenase (GAPDH) assay. The GraphPad prism software (GraphPad, San Diego, California, USA) was used to plot the standard curve and calculate the concentration factors.

### Measurement of GAPDH Gene Expression

To quantify the GAPDH mRNA levels in the experiments, the cell lysates of treated cells with the least synergistic doses and untreated control cells were collected at regular intervals (6, 12, 18, and 24 h) and frozen at −86°C until they were used. The GAPDH enzyme mRNA level was determined by using a QPCR assay.

All RNA in the cell lysate was isolated using a Magnesia® total RNA extraction kit (Anatolia Geneworks, Turkey) according to the manufacturer's protocol. The fully automated Magnesia Extraction machine (Anatolia Geneworks, Turkey) was used to extract the RNA from the cell lysate. The yield was quantified using the Biodrop machine (Biochrom, UK).

The isolated RNAs were reversed transcribed to produce double-stranded cDNA by the reverse transcriptase polymerase enzyme using KAPA SYBR FAST One-Step qRT-PCR universal kit (Kapa Biosystems, Cape Town, South Africa), and they were measured in real-time PCR using the MX3005 Real-Time PCR machine (Agilent Technologies, USA). Specific primers were made for GAPDH by Integrated DNA Technologies (Brussel, Belgium) to detect the mouse GAPDH gene in the AMN3 cells: 5′ ATCACTGCCACCCAGAAGACTG 3′ (sense) and 5′ AGGTTTTTCTAGACGGCAGGTCAG 3′ (antisense) for human AMJ13 cells and 5′ GGAGAGTGTTTCCTCGTCCC 3′ (sense) and 5′ TTTGCCGTGAGTGGAGTCAT 3′ (antisense).

The amplification conditions of the reaction that was used for human and mouse GAPDH amplification were as follows: 42°C for 5 min, denaturation at 95°C for 10 min, and amplification in 40 cycles, each of which at a denaturation temperature of 95°C for 3 s, annealing temperature at 60°C for 20 s, and elongation temperature at 72°C for 20 s. Controls were included in each run of the real-time PCR assay; for each primer pair, one sample with no cDNA (containing only RNase free water) was included.

The results of each sample were analyzed using a relative quantification to compare the difference between the sample and the control. The mean CT values of the genes were calculated in each sample (a duplicate replication for each sample) and used to normalize the expression levels by applying the ΔΔCT method described previously (Livak and Schmittgen, 2001).

### Pyruvate Assay

The levels of Pyruvate were measured by using colorimetric assay kits, according to the manufacturer's instructions (ElabScience, USA). The cells lysates and conditioned media of treated cells with the least synergistic doses and untreated cells were collected after 48 h. The OD values were measured of each sample at 505 nm in spectrophotometer.

### *In vivo* Experiments

#### Animals

All animals were maintained according to the guidelines of the Iraqi Center for Cancer and Medical Genetic Research (ICCMGR) in the animal house facility. All experimental studies were approved by the Institutional Animal Care and Use Committee of Mustansiriyah University, College of Science and ICCMGR.

#### Animal Tumor Model

The murine mammary adenocarcinoma tumor (AN3) used in the current experiment was described previously (Al-Shamery et al., 2008). The AN3 tumor line was derived from a spontaneously arising mammary tumor in an albino Swiss mouse. The AN3 tumor line is maintained by continuous transplantation in inbred syngeneic mice. This cell line has the same origin of the AMN3 cell line that is used in *in vitro* experiments.

#### Experimental Design

Tumors were established by inoculating AN3 cells (10^6^/100 μl per site) into the right flanks of 6–8-week-old female Swiss Albino mice (ICCMGR, Animal House Unit, Baghdad, Iraq). When the tumors nodules reached 0.5–1 cm in diameter, the animals were randomly divided into four groups of five: The first group of mice received intratumoral (IT) injections of NDV Iraqi AMHA1 isolate at 256 HAU in 100 μl PBS; the second group received 2-DG intraperitoneally with a total dose of 2,500 mg/kg of 2-DG alone (one injection of 500 mg/kg/24 h for 5 days); the third group received a combination of both. The mice in the fourth group of controls were left untreated on day 10 post implantations. After 30 days, the animals were anesthetized and then sacrificed using a lethal dose of diethyl ether.

#### Assessment of Anti-tumor Efficacy

The tumor diameters were measured every third day, and their sizes were measured using calipers. The tumor volume was calculated (product of 0.5 × length × width × width) (Al-Shamery et al., 2011) as mean ± SEM for each group. The mice were sacrificed when the tumor burden reached a volume of ~10% of their body weight.

To calculate the tumor growth, the tumor volume was normalized to the volume of each tumor at time zero, which was the time point at which the treatment was initiated. Tumor growth inhibition (TGI) (Phuangsab et al., 2001) was measured twice weekly during the evaluation period by the following formula:

(1) $$\begin{array}{l} \text{GI}\%\;=\frac{\text{tumor volume of untreated group - tumor volume of treated group}}{\text{tumor volume of untreated group}}\text{ x100} \end{array}$$

A tumor growth inhibition >50% was considered meaningful.

#### Statistical Analysis

The MTT assay and the RT-PCR data (i.e., average means after determining the ΔΔCT values) were statistically analyzed using the one-way analysis of variance test (ANOVA) in GraphPad Prism (GraphPad Software, Inc. San Diego, California). The standard deviation of the mean was considered significant at *P* = 0.05. Unpaired *t*-tests were used for comparison between groups and *p* < 0.05 were considered significant. One-way ANOVA with Tukey's multiple comparisons test were performed to determine significance of AO/PI and Pyruvate assays.

Statistical analyses for *in vivo* study were performed with GraphPad Prism (GraphPad Software Inc.). One-way ANOVA analysis of variance tests were used for statistical comparison between three or more groups. Data in graphs are shown as mean ± S.D.

## Results

### *In vitro* Anti-tumor Activity of 2-DG–NDV

**Figure 1:**
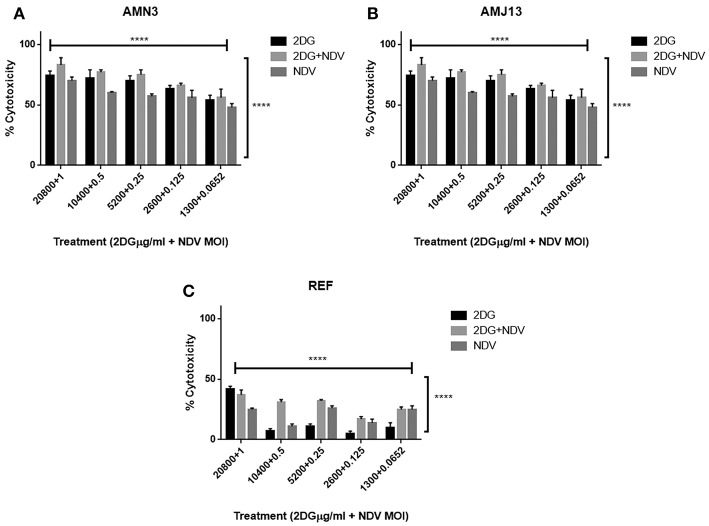
All treatment modalities (2DG, NDV, and 2-DG-NDV) induced significant proliferation inhibition and effectively reduced the viability of cancer cells without cytotoxicity in normal cells. **(A)** AMN3 cells cytotoxicity; **(B)** AMJ13 cell-line cytotoxicity; **(C)** No cytotoxicity of the three treatment modalities on REF normal cells as the killing effect was <50%. ^****^means highly significant (*P* ≤ 0.0001).

To evaluate the therapeutic efficacy of 2-DG–NDV and its potential cytotoxicity, MTT cell viability assays were carried out in the cancer cell lines (mouse AMN3 and human AMJ13) and normal cells (REF). As shown in Figures 1A,B, the results indicated that the all treatment modalities effectively reduced the viability of the cancer cells significantly (more than 50% at all concentration tested) and had inconsiderable cytotoxicity in the normal cells (all below 50% cytotoxicity) (Figure 1C).

### Chou-Talalay Analysis and Synergism Determination

**Figure 2:**
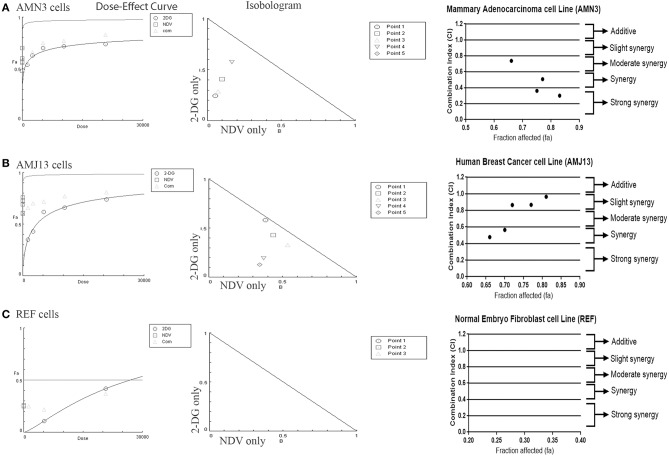
Illustration of Dose–effect curves, normalized isobologram of non-constant combination ratios. Moreover, data shown as fraction affected (fa) vs. CI plots. CI < 1 indicate synergy, CI ¼ 1 indicate additive, and CI > 1 indicate an antagonistic interaction. **(A)** Effect on AMN3 cell line and all of the points showing synergy; **(B)** First point showing nearly additive effect while the rest of the point showed synergism for the cytotoxic effect on AMJ13 cells; **(C)** the point tested showed strong antagonism on the normal REF cell and neglected cytotoxic effect as all concentration used failed to induce near 50% cytotoxicity. Points are the combination of NDV [MOI] and 2-DG [μg] (1 20,800), (0.5 10,400), (0.25 5,200), (0.125 2,600), and (0.0652 1,300). The data shown are representative of 3 separate experiments.

The possible interactions in virotherapy by the NDV Iraqi AMHA1 strain and 2-DG as an anti-breast cancer therapy were evaluated. As Synergism/Antagonism quantification described as mass-action law issue (determined by the CI values), and not a statistical issue (not determined by the p values) (Chou and Martin, 2005). The combination of NDV and 2-DG were evaluated using Chou-Talalay equations. A CI <0.9 was considered synergistic, a CI between 0.9 and 1.1 was considered additive, and a CI >1.1 was considered antagonistic (Chou, 2006). In the combination of NDV and 2-DG, the Chou-Talalay CI was 0.37325 in the AMN3 cell lines and 0.63497 in the AMJ13 cell line as shown in Figures 1A,B of combination index plots. The plots of Chou–Talalay show moderate to strong synergistic cell killing between 2DG and NDV (combination) in AMN3 and AMJ12 cells. These results demonstrated the synergistic cytotoxicity of almost all the concentrations tested (Figures 2A,B; Table 1). Moreover, NDV and 2-DG was antagonistic when tested on embryo fibroblasts cells at all doses tested (Figure 2C).

**Table 1:** AMN3, AMJ13, and REF cells were treated with NDV or 2-DG alone or a combination at the indicated concentrations for 72 h, and an MTT assay was performed.

**Points**	**NDV [MOI]**	**2-DG [μg]**	**CI**	**Effect**
**A. AMN3 cells**				
1	1	20,800	0.30056	Synergism
2	0.5	10,400	0.50725	Synergism
3	0.25	5,200	0.36134	Synergism
4	0.125	2,600	0.73701	Moderate synergism
5	0.0652	1,300	1.45873	Antagonism
**B. AMJ13 cells**				
1	1	20,800	0.96494	Nearly additive
2	0.5	10,400	0.86616	Slight synergism
3	0.25	5,200	0.86410	Slight synergism
4	0.125	2,600	0.56462	Synergism
5	0.0625	1,300	0.47712	Synergism
**C. REF cells**				
1	1	20,800	20343.6	Very strong antagonism
3	0.25	5,200	3.98300	Strong antagonism
5	0.0625	1300	4.91389	Strong antagonism

*CI was calculated by CompuSyn software. **(A)** CI values of NDV in combination with 2-DG on AMN3 cancer cells; **(B)** CI values of NDV in combination with 2-DG in AMJ13 cancer cells; **(C)** CI values of the same combinations on non-cancer cells (REF), effects below 10% were not included due to recommendation of CompuSyn Software. Effect described according to (36)*.

### Apoptotic Cell Death Evaluation Using Acridine Orange/Propidium Iodide

**Figure 3:**
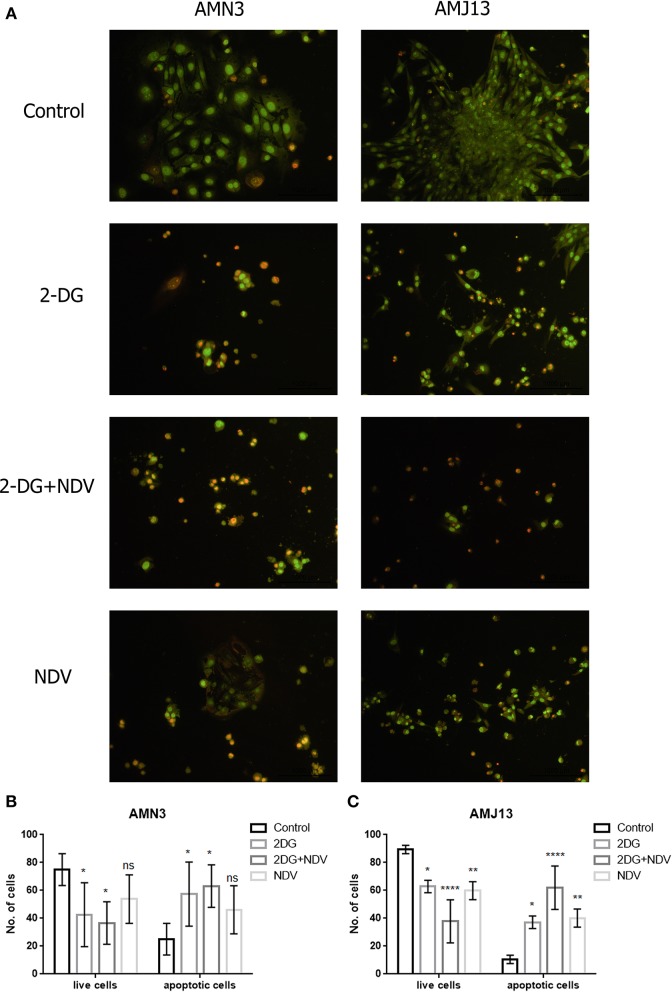
2DG-NDV induces apoptosis in breast cancer cell lines: **(A)** Acridine orange and propidium iodide staining of AMN3 and AMJ13 cancer cells indicated that 2DG-NDV induce apoptosis as evidenced red and orange stained cells, Untreated control cells emitting green fluorescence. **(B)** The number of live and apoptotic cells in AMN3 mammary adenocarcinoma cell line were quantified and analyzed. Combination therapy using 2DG-NDV showing the significant increase in apoptotic cells at 48 h. **(C)** 2DG-NDV treated AMJ13 breast cancer cells expressed significant apoptotic cell death. ^ns^*P* > 0.05, ^*^P ≤ 0.05, ^**^P ≤ 0.01, ^***^P ≤ 0.001, ^****^P ≤ 0.0001.

The ability of 2-DG–NDV apoptosis induction of cell death in breast cancer cells was confirmed via acridine orange/propidium iodide double stain. The results showed in Figure 3 that untreated control cells generally viable non-apoptotic cells emitting green fluorescence (Figure 3A). However, 2-DG–NDV treated cancer cells were emitting red fluorescence more than monotherapies significantly (Figure 3A). Apoptotic cells usually emitting red or orange fluorescence. These results were confirmed by quantitative analysis that showed significant increase in apoptotic cells in 2-DG–NDV treated cancer cells (Figures 3B,C).

### ELISA Assay Analysis Indicates Strong GAPDH Enzyme Level Reduction in Both Breast-Cancer Cell Lines Treated by 2-DG-NDV Combination Therapy

**Figure 4:**
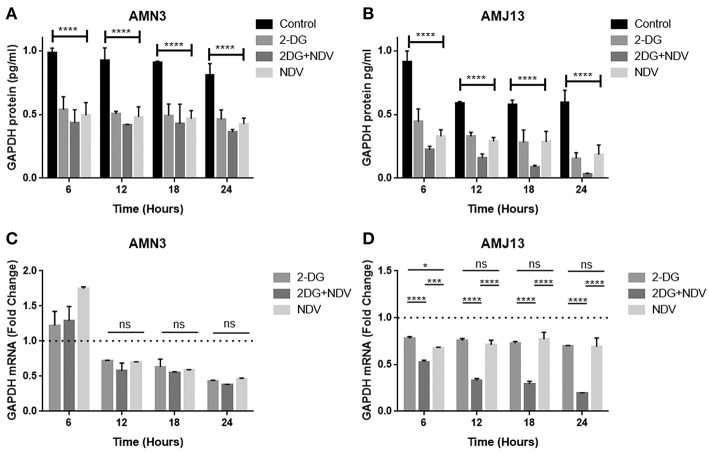
Exposure of AMN3 and AMJ13 cells to the least synergistic dose of 2DG–NDV for 6, 12, 18, and 24 h and measuring GAPDH protein and mRNA expression in the treated and untreated cancer cells. **(A)** GAPDH protein concentrations reduced significantly in treated AMN3 cells as measured by specific quantitative ELISA assay and analyzed by one-way ANOVA in compare to control cells, all treatment modalities were successful to induce this reduction. **(B)** AMJ13 human breast cancer cells were more sensitive to treatment modalities as GAPDH protein showed significant reduction and the 2DG-NDV induce more decrease in the GAPDH concentration. **(C)** GAPDH mRNA level of expression in AMN3 cells was downregulated after 12 h of exposure by all treatment modalities while the 2DG-NDV showed higher non-significant downregulation to the GAPDH mRNA. **(D)** Human AMJ13 cells were also more sensitive to the treatment modalities that induced GAPDH mRNA downregulation at all time measured. Combination therapy of 2-DG–NDV showed significant downregulation in compare to single agents. Asterisks denote statistical significance (*P* < 0.05).

In the current study, we quantified and analyzed the GAPDH protein expression in both the AMN3 cell line and the AMJ13 cell line. The ELISA assay determines protein identification, quantification, and data quality with sensitivity and repeatability. The GAPDH enzymes were evaluated for the comparison of treated and untreated cells at 6, 12, 18, and 24 h (Figures 2A,B). We identified a significant reduction in the GAPDH protein expressed in all the (2DG, NDV, and 2-DG–NDV) treated cells compared with the untreated cells in the control group, which were collected at 6, 12, 18, and 24 h. The results showed that the GAPDH protein concentration was significantly reduced in the combination of 2-DG-NDV used to treat the AMN3 cells and it is more significant than single treatment modalities (Figure 4A). Human breast cancer AMJ13 cells were more sensitive to the therapeutic agents especially to the combination of 2DG-NDV (Figure 4B). NDV and 2-DG alone were also reduced GAPDH concentration to lesser degrees. These results suggest that NDV may play an important role in glycolysis metabolism inhibition in cancer cells. Moreover, the inhibition rate increased significantly with time.

### 2-DG–NDV Induced GAPDH Gene Downregulation

The exposure of the AMN3 and AMJ13 cells to the least synergistic dose of 2DG–NDV at 6, 12, 18, and 24 h led to a clear significant reduction in GAPDH gene expression compared to the untreated control cells (Figures 4C,D). We observed the downregulation of the GAPDH gene as early as 12 h after treatment in mouse mammary adenocarcinoma AMN3 cells while it was earlier at 6 h after exposure in human breast cancer AMJ13 cells. To validate the protein downregulation shown by the quantitative ELISA assay, we chose QPCR to assess the expression at various time points following the 2DG–NDV treatment in the cancer cell lines and to confirm that the decrease in mRNA led to the reduction in protein levels. Using real-time PCR (RT-PCR), we confirmed that GAPDH genes were transcriptionally downregulated by 2DG–NDV, NDV alone, and 2DG alone. Consistent with the decrease in GAPDH protein levels determined by the ELISA assay induced by the treatment modalities, the GAPDH mRNA levels also decreased significantly as tested by ANOVA multiple comparison in both treated cell lines AMN3 and AMJ13 when comparing the treated and untreated cells. In cells exposed to the 2-DG–NDV combination, GAPDH protein inhibition was higher than single modalities, constantly GAPDH mRNA inhibition by combination treatment was significantly higher than single treatment modalities as well in both cell lines. Table 2, summarize values for the percentage of inhibition for the GAPDH protein and mRNA after treatment in both treated cell lines AMN3 and AMJ13 cell lines.

**Table 2:** Values for the percentage of inhibition for the GAPDH protein and mRNA after treatment in **(A)** AMN3 and **(B)** AMJ13 cell lines.

**Time h**	**2DG inhibitory effect %**		**Com inhibitory effect %**		**NDV inhibitory effect %**	
	**Protein %**	**mRNA %**	**Protein %**	**mRNA %**	**Protein %**	**mRNA %**
**(A) AMN3**						
6	40.50	16	51.76	40	45.67	20
12	41.80	29	53.90	42.50	46.95	29
18	45.67	38	56.45	48	48.98	43
24	48.98	56	59.86	62	53.49	55
Average	44.24	35	55.49	48	48.77	37
**(B) AMJ13**						
6	51	22	75	45	63.40	30
12	45	26	73	67	51	30
18	51	29	84.20	71.40	51	24
24	74	23	95	79	70	26
Average	55	25	82	66	58.85	28

### 2-DG–NDV Efficiently Inhibits Glycolysis in the Treated Breast Cancer Cells

**Figure 5:**
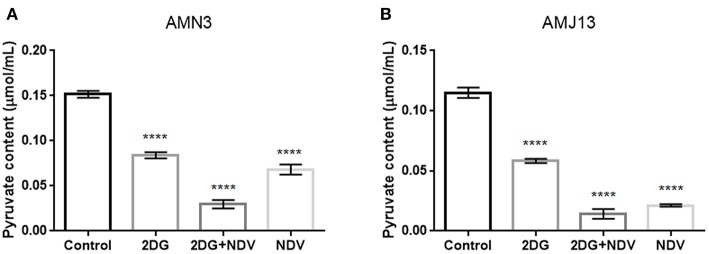
Further investigation to the mechanism by which 2-DG–NDV inhibit breast cancer cell proliferation was done through examination whether 2-DG–NDV efficiently decrease cancer cell glycolysis product pyruvate and compared to monotherapies. Notably, 2-DG–NDV had significant suppressant effect on the levels of pyruvate in both cancer cell lines **(A)** measurement in mouse mammary adenocarcinoma AMN3 cells. **(B)** Measurements in human breast cancer cells AMJ13. ^****^means highly significant (*P* ≤ 0.0001).

Further investigation to the mechanism by which 2-DG–NDV inhibit breast cancer cell proliferation was done through examination whether 2-DG–NDV efficiently decrease cancer cell glycolysis product pyruvate and compared to monotherapies. Notably, 2-DG–NDV had significant suppressant effect on the levels of pyruvate in both cancer cell lines (Figures 5A,B).

### Therapeutic Experiment on Mouse Mammary Adenocarcinoma Tumor Model (AN3)

**Figure 6:**
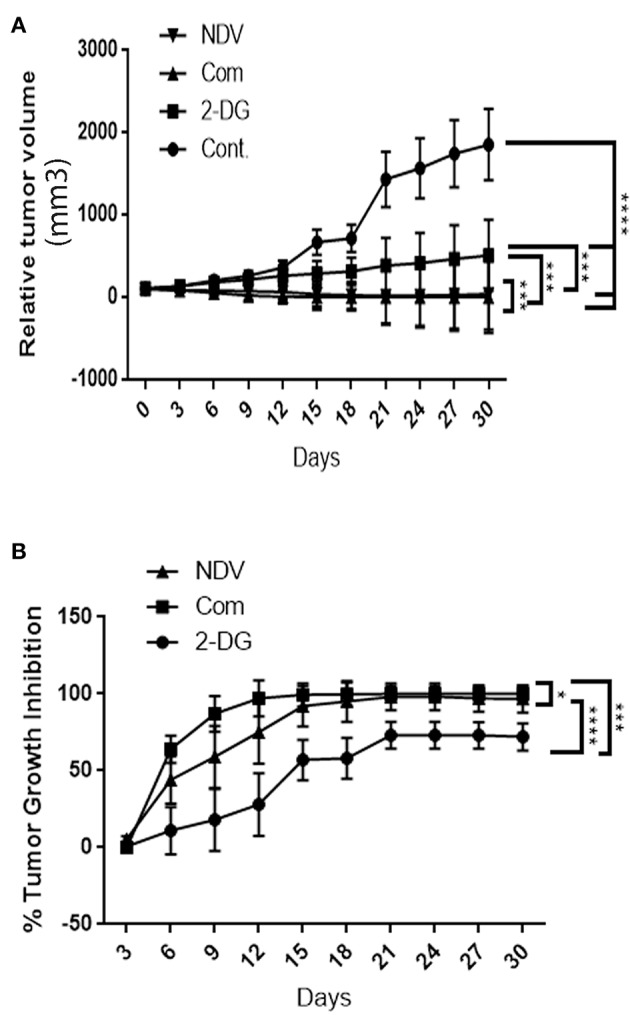
*In vivo* tumor growth rate in albino Swiss mice. When the tumor nodules reached 0.5–1 cm in diameter, the animals were randomly divided into four groups of five. The first group received intratumoral (IT) injections of NDV Iraqi AMHA1 isolate at 256 HAU in 100 μl PBS; the second group received 2DG intraperitoneally with a total dose 2,500 mg/kg of 2DG alone (one injection of 500 mg/kg/24 h for 5 days); the third group received the combination of both. The control group was left untreated on day 10 post implantation. **(A)** Relative tumor volumes. In response to the combination therapy, the 2-DG–NDV treatment group achieved significant tumor size reduction (*P* < 0.05) when compared to single treatment modalities. All treatment modalities showed significant reduction in tumor size. **(B)** Combination therapy group induced significant tumor growth inhibition rate (100%) at the end of the experiment, followed by NDV alone (96.8%). 2DG alone induced a growth inhibition of 72%. Growth inhibition rates are shown for each point measured during the study period. The reduction in combination group is more than in NDV alone but the difference is not significant. Asterisks denote statistical significance (*P* < 0.05).

To examine the hypothesis that NDV viral toxicity increases *in vivo* in response to the 2DG treatment, we injected albino Swiss mice with AN3 tumor cells. The cells were injected under the skin to allow for precise tumor measurement. When the tumor nodules reached 0.5–1 cm in diameter, the animals were randomly divided into four groups of five, as described in the methods section. As shown in Figure 6A, the relative tumor volumes were plotted over a 30-days period. All treatment modalities induced significant (*P* < 0.0001) decrease in tumor volume in compare to the control untreated group. In response to the combination therapy, the 2-DG–NDV treatment group achieved significant tumor size reduction (*P* < 0.0003, *P* < 0.0006) when compared to 2DG and NDV treatment groups, respectively. Furthermore, the combination therapy group induced the highest rate of tumor growth inhibition (100%) at the end of the experiment, followed by the NDV group (96.8%). The 2DG group had the lowest rate of growth inhibition (72%) as shown in Figure 6B. In the untreated control group, the tumors continued to grow throughout the experiment.

## Discussion

The present work compared the effects of our novel combination therapy of DNV and 2DG on *in vitro* mouse and human breast-cancer cell lines and used an *in vivo* mouse model to determine the effectiveness of the combination therapy. Breast cancer causes highly malignant tumors, resulting in poor prognoses (Soerjomataram et al., 2008). The currently established breast cancer chemotherapeutics and radiotherapy have limited effectiveness (Hickey et al., 2013). Ciavardelli et al. (2014) showed that breast cancer stem cells rely on fermentative glycolysis and are sensitive to glycolysis inhibitors such as 2DG. The results of the current work provide evidence that combining glycolysis inhibitor (using 2-DG) with oncolytic virotherapy (NDV) could synergize *in vitro* to induce tumor cell death and inhibit tumor growth in an immunocompetent mice model that had been implanted with mammary adenocarcinoma tumor. This combination of agents was more efficient than either agent administrated alone in inducing the inhibition of the glycolysis pathway in cancer cells.

The aim of this study was to evaluate the increase in the sensitivity of cancer cells to oncolytic virotherapy using 2DG and the synergistic combinations of different concentrations that enhanced the cytotoxic effect. An MTT assay was conducted using several different concentrations of 2DG and NDV. The results indicated that the NDV, 2DG and combination of 2-DG–NDV effectively reduced the viability of the cancer cells and showed insignificant cytotoxicity in the normal cells. The combinations of NDV and 2DG were evaluated using the Chou-Talalay equation. Quantification of synergism and antagonism is a mass-action law issue that is determined by the combination index values, not by statistical issue that can be determined by the p values (Chou and Martin, 2005). The results demonstrated synergistic cytotoxicity in almost all the concentrations tested on the cancer cells. Moreover, the combination of NDV and 2DG has no effect when tested on normal embryo fibroblasts cells at most doses tested which prove safety.

Several previous studies reported that 2DG might enhance anti-breast cancer effect of other chemotherapeutics (Gupta et al., 2009; Aghaee et al., 2012; Cheng et al., 2012; Zhang et al., 2014). However, our current study is the first to demonstrate the presence of synergism between 2DG and oncolytic NDV. To explain this synergistic enhancement of the anti-tumor action, we need to address the fact that cancer cells depend on glycolysis rather than oxidative phosphorylation even under aerobic conditions, which results in the high glucose uptake of cancer cells compared to normal tissues to produce energy and support the metabolic function, which is known as the Warburg effect (Warburg, 1956). Our selective therapy targeted the glucose metabolism as a site of intervention by 2DG (glycolysis inhibitor). Another method of using glycolysis inhibition as a cancer treatment is to identify agents that could further sensitize tumor cells under glycolytic inhibition (Li et al., 2008). Hence, based on the results of this study, we propose that NDV may further interfere with the glycolysis pathway by different mechanisms that amplify the glycolysis inhibition, leading to the starvation of cancer cells. Deng et al. (2014) suggested that NDV may reduce the activity of the host's glycolytic pathway by decreasing phosphoglycerate kinase (PGK) expression in the NDV-infected PBMCs. PGK is a glycolytic enzyme that plays an important role in cellular energy production, and it forms part of the glycolytic pathway (Wang et al., 2010). The AO/PI apoptosis assay showed that 2DG-NDV is the most effective treatment to induce apoptotic cell death in both cell line tested. NDV Iraqi strain induce increase in caspase-3 and DNA fragmentation leading to cell death (Al-Shammari et al., 2014d; Al-Shammary et al., 2014).

To demonstrate the proposed mechanism of the combination, we studied GAPDH, which is an important key enzyme in the glycolysis pathway. GAPDH is considered one of the most important enzymes in cell energy metabolism. It catalyzes the sixth step, specifically by catalyzing the reversible conversion of glyceraldehyde-3-phosphate (G-3-P) to 1,3-diphosphoglycerate (Zhang et al., 2015). The results showed that GAPDH protein as well as mRNA were downregulated in combination 2-DG–NDV-treated AMN3 and AMJ13 cancer cells compared with the untreated control cells. NDV alone and 2-DG alone were also downregulating GAPDH but to lesser degrees. Interestingly, the expression levels of GAPDH were downregulated in the NDV-treated cells more than in the 2-DG-treated cells at some time points tested in both cancer cell lines, this result suggests that NDV may play an important role in inhibiting cancer cells glycolysis metabolism. Using a quantitative ELISA and real-time PCR assays, we confirmed that the GAPDH gene was transcriptionally downregulated by the combination 2DG–NDV. The ELISA assay showed a decrease in GAPDH protein levels, which was consistent with the results of the QPCR assay. The GAPDH mRNA levels also decreased in both cell lines. The significant downregulation of GAPDH by the combination therapy could explain the notable enhancement of cancer cell cytotoxicity. It also suggests that NDV and/or the combination induced the direct inhibition of GAPDH expression. Ganapathy-Kanniappan and Geschwind (2013) found that in GAPDH inhibition, in addition to the effects on glycolysis and ATP production, a multipronged effect triggered a cascade of events that eventually led to cancer cell death. These events were initiated by accumulations of glucotrioses, such as dihydroxy acetone phosphate, and glyceraldehyde-3-phosphate, within the cell because they could not be metabolized. The partial degradation of glucotrioses results in the formation of cytotoxic metabolites such as methylglyoxal, which in normal conditions enters the glyoxalase system to be detoxified. However, in the presence of oxidative stress induced by 2DG (Simons et al., 2009) and NDV (Al-Shamery et al., 2012; Cheng et al., 2016) treatment and glutathione (GSH) depletion due to the accumulation of reactive oxygen species (ROS), the activity of glyoxalase 1 (Glo1) declined, leading to the accumulation of methylglyoxal, which has a direct cytotoxic effect on cancer cells (Thornalley and Rabbani, 2011). Therefore, the inhibition of GAPDH was shown to be major anti-tumor mechanism and a novel target. Phadke et al. (2011) reported that GAPDH depletion accelerated a cellular senescence phenotype of human lung carcinoma cells. They examined the effects of GAPDH knockdown on carcinoma cells, which established proliferation arrest, changes in morphology, and more than the 2-fold up-regulation of senescence-associated genes resulting from compromised glycolysis and energy crises. GAPDH is very promising target for the treatment of a wide range of tumors, in contrast to other specific anticancer agents, such as Herceptin, which is used in the treatment of HER2-positive breast cancer only (Krasnov et al., 2013). 2DG-NDV suppressed the glycolysis pathway as revealed by the decrease of glycolysis intermediates such as pyruvate. Its shown that 2DG reduce glycolysis products, G-6-P, pyruvate and lactic acid in hepatocarcinoma cells with no effect on normal liver cells (Anantharaju et al., 2017).

To confirm the *in vitro* results, we conducted an *in vivo* study on immunocompetent mice transplanted with mouse mammary adenocarcinoma cells. The results of the *in vivo* study indicated that the anticancer effect of 2-DG–NDV was higher than that of the free 2-DG and the free NDV, which confirmed the *in vitro* results. The reduction in combination group is more than in NDV alone but the difference is not significant. The oncolytic effect of the Iraqi NDV strain was previously proved to have several anti-tumor mechanisms. One of these mechanisms are apoptosis induction in the infected tumor cells both *in vitro* and *in vivo* through the mitochondrial pathway. However, there was an association of an extrinsic pathway to a lesser degree *in vivo* (Al-Shammari et al., 2015a). NDV was shown to stimulate a specific anti-tumor immune response (Al-Shamery et al., 2009) and induce endoplasmic reticulum stress (Al-Shamery et al., 2012). These mechanisms, in addition to the direct cytolytic effect, were secondary to virus replication (Al-Shammari et al., 2010). Moreover, 2DG was shown to have an anti-tumor effect *in vitro*, which was improved in combination with cytotoxic drugs *in vivo* and was found to sensitize chemotherapeutic agents to breast cancer stem cells (Cheng et al., 2012; Ciavardelli et al., 2014). These mechanisms and the current *in vitro* results showing GAPDH downregulation explained the *in vivo* anti-tumor synergism. When synergized with chemotherapeutic agents, both NDV and 2-DG were found to increase significantly the anti-tumor activity in immunocompetent mice (Al-Shammari et al., 2011; Bénéteau et al., 2012). Moreover, both 2DG and NDV were shown to reduce the activity of the glycolytic pathway (Aft et al., 2002; Deng et al., 2014). Li et al. (2015) found that the attenuated measles vaccine virus shifted the cellular metabolism to a high rate of glycolytic adaptation, which was efficiently targeted by dichloroacetate (DCA) (an inhibitor of glycolysis), leading to marked anti-tumor activity both *in vitro* and *in vivo* without any toxicity observed in normal cells. Moreover, 2-DG showed antiangiogenic effects by interfering with N-linked glycosylation at 500 mg/kg, the same dose used in our study (51). NDV was also shown to downregulate several angiogenic factors, including the vascular endothelial growth factor (VEGF), intra-cellular adhesion molecule 1 (ICAM-1), epidermal growth factor (EGF), and basic fibroblast growth factor beta (FGF-B) (52). By combining these two agents, we may have promoted extensive anti-angiogenic activity. Furthermore, 2-DG was reported to be an anti-virus agent because it was shown to interfere with N-linked glycosylation and reduce protein synthesis (53, 54). However, (55) recently reported increased N-linked glycosylation in cancer cells by certain doses of 2-DG treatment, which could explain why there is several phases of synergism at different doses in the current study. Interestingly, most studies about the effects of 2-DG on cellular glycosylation were done within a very short time frame (a few hours) after the application of 2-DG. In contrast (55), observed 2-DG effects after an application period of 48 h, which agrees with our results of the longer application period of 72 h, which is relevant for clinical applications.

In conclusion, the results of our study support a novel therapeutic strategy that provides a rationale for combining virotherapy using the oncolytic NDV Iraqi strain with 2-DG in a treatment modality that targets the cancer cell metabolism. This study is the first to report the demonstration of a possible mechanism achieved through targeting the GAPDH enzyme, which produced a very significant anti-tumor response. The strategy could be used in clinical cancer therapy.

## Ethics Statement

All animals were maintained according to the guidelines of the Iraqi Center for Cancer and Medical Genetic Research (ICCMGR), Mustansiriyah University, in the animal house facility. All experimental studies were approved by the Institutional Scientific Committee of Mustansiriyah University, College of Science and ICCMGR.

## Author Contributions

AA-S and AA study concept and design. AA-S and ZA performed experiments. AA-S, AA, ZA, and NY data analysis. AA-S, ZA, and AA drafted manuscript draft. AA-S, AA, and NY approved of manuscript.

### Conflict of Interest

The authors declare that the research was conducted in the absence of any commercial or financial relationships that could be construed as a potential conflict of interest.
